# Case of Uterine Hemorrhage, Successfully Treated by the Operation of Transfusion

**Published:** 1825-11

**Authors:** Edward Doubleday

**Affiliations:** Fellow of the London Medical Society, &c.


ART. IV.—*Case of Uterine Hemorrhage, successfully treated by the Operation of Transfusion.* By EDWARD DOUBLEDAY, Esq. M.R.C.S. and Fellow of the London Medical Society, &c.

MRS. COCKLIN, æt. twenty-nine, a woman of robust constitution, residing at 59, Brunswick-street, Stamford-street, was delivered of a female child, by my friend, Mr. George Franks, on Wednesday, September 28, at a quarter past four A.M. Nothing particular occurred during the birth of the child. The usual means were resorted to, to cause the expulsion of the placenta, but without success. After the lapse of about two hours, hemorrhage came on to an alarming extent. The hand was now introduced into the cavity of the uterus; and the placenta, which was strongly adherent to its fundus, was extracted as speedily as possible.

At this time Mr. G. Franks requested my attendance. At the period of my arrival, I found the uterus contracted upon the placenta, which was now being brought away.

Before and during the extraction of the placenta, so much blood was lost that the woman was reduced to syncope; and her appearance was such that, on my entering the room, I feared speedy dissolution would ensue. The pulse could not be felt at the wrist; the countenance was exsanguineous; lips pale; nostrils pinched; sight indistinct; excessive restlessness; breathing hurried, accompanied with frequent sighing; and the body was covered with cold clammy perspiration. I gave her a teacupful of brandy (about six ounces); after which the pulse became just distinguishable. Brandy was repeated at short intervals; and, in addition to this stimulus, carbonate of ammonia and laudanum were administered. These measures were employed vigorously for half an hour, but their influence was very transitory. The pulse was sometimes perceptible and very rapid, and at others could not be felt. No real advantage being obtained by these means, it appeared to me that the only remedy left, by which it was probable that the life of the patient could be preserved, was the operation of transfusion.

I therefore immediately set off for Dr. Blundell, leaving Mr. Franks in care of the patient. I fortunately found the Doctor at home, and he politely accompanied me to her residence without delay.

We found the woman certainly not worse; but, after a deliberate investigation of the present state of the pulse, aspect of the countenance, and the quantity of blood lost, it was Dr. Blundell's decided opinion, in which I fully concurred, that the probabilities against her recovery, without transfusion, were so strong as reasonably to demand the operation. The operation was proposed to the husband, who not only agreed to it, but also to furnish us with the necessary supply of blood.

Dr. B. commenced the operation by exposing the median cephalic vein, which the patient resisted to an astonishing degree. The opposition which was offered on the part of the patient, induced the Doctor to abandon the operation for the time: not, however, with the expectation that she would ultimately recover.

The idea of being left to witness the death of this poor woman, without making the attempt to save her life by the operation of transfusion, operated so powerfully upon my mind as to induce me to request the Doctor to leave his syringe, &c. with a hope that, when the woman was *in articulo mortis*, I should be able to perform it.

Mr. G. Franks was again left in care of the patient, with directions to continue the stimulants, &c. &c. About half-past two o'clock P.M., *being upwards of six hours after the hemorrhage had stopped*, I received a note from him, stating that she was *sinking very fast*, and that he did not believe she would be alive by the time I could get there. I immediately proceeded to the spot, and found the patient all but gone. At this time twenty ounces of brandy, 160 drops of laudanum,* and a considerable quantity of ammonia, had been given; besides the yolks of three eggs beaten up in brandy, and some beef-tea and gruel.

As no time was to be lost, and as the friends were anxious that the operation should be tried, I at once proceeded to pass a blunt needle under the vein which had been already exposed by Dr. B. When this was accomplished, I made an opening into it, large enough to admit the beak of the syringe. Mr. F. placed his finger upon the vein, and gently pressed it upon the needle, in order to prevent any loss of blood. A vein in the arm of the husband was now opened freely, and the blood allowed to flow into a conical drinking-glass; and, as it flowed

---

* The laudanum was given within the first two hours after the flooding had ceased, with a view to allay the extreme restlessness; and apparently with benefit.

into the glass, I absorbed it into the syringe, taking care to keep the beak at the bottom, which was covered with about half an ounce of blood. The object of this precaution was to prevent any bubbles of air from being absorbed with the blood; but, to be quite positive on this head, I placed the syringe with the beak perpendicularly above the handle,—that is, with its nosle upwards,—and forced a little blood through it, before I introduced it into the vein. The beak of the syringe being carefully introduced into the opening made in the vein, the blood was gently forced in, in a direction towards the heart. As the syringe was emptied, *the pulse rose.* The syringe was refilled, but not before the small quantity of blood which was left in the glass was thrown away: and, as this second quantity was injected, *the pulse became broader,* and the appearance of the lips and countenance much improved. Another syringeful was thrown in, (making six ounces,) in the same way, with very marked improvement in the condition of the patient. So much was she recovered, that, to use her own language, she exclaimed "I am as strong as a bull!" As the fourth syringeful was being injected, she stated she felt the blood going up the veins. This injection produced additional improvement in her aspect and in the pulse, which was such as indicated considerable power in the system; and she stated that she felt quite recovered.

I here hesitated whether it would not be better to stop the process: however, as there was no unfavourable symptom, and as the patient had improved upon the injection of every additional quantity, I determined to go on till something should occur which might induce me to believe that the operation should not be proceeded in further. I accordingly threw in *three* more syringefuls in the same way, (making fourteen ounces,) with continued improvement in the power of the pulse; which now led me to believe that, notwithstanding the *copiousness* of the *flooding*, the *large vessels* were distended as much as at any time during health. She at this period complained of a little pain over the left eye, which induced me to discontinue the process of injection, thinking that as much blood had been thrown in as the woman could bear with impunity. The opening in the vein was therefore closed in the usual way.

*The pulse, some time previous to the operation, when it could be numbered, was* 140, *but was now reduced to* 104. *A quarter of an hour after the operation, it was* 98; *in half an hour,* 90. *The pulse, for the most part, was strong, soft, and full, but somewhat irregular, and continued so for upwards of two hours. At about the expiration of an hour after the blood was injected, she sat up, and assisted the nurse in undressing and in making herself more comfortable, as if nothing had occurred beyond what is observed in ordinary cases.*

Half-past six P.M. (three hours after the operation.)—Pulse 94, strong, and full; skin hot; tongue becoming furred; thirst considerable; has had some little uterine pain; bowels constipated. These symptoms might be attributed, in a great measure, to the influence of the brandy, ammonia, and opium. I now ordered a purgative enema, which was immediately injected.

Half-past eight P.M. (five hours after the operation,) I again saw her. She had slept a little since my last visit. The enema had produced two copious evacuations. Pulse 120; skin perspiring freely; tongue moist. Has taken nothing into the stomach since the operation. At this time she felt restless, and had some pain in the hypogastric region, which induced me to order sixty drops of tincture of opium.

Half-past eleven P.M. (eight hours after the operation.)—*Nothing has been taken into the stomach since the operation was performed.* Skin covered with copious perspiration. Has had some refreshing sleep.—Ordered an aperient.

Thursday, six o'clock A.M.—Slept comfortably through the night. Pulse varying from 96 to 100; skin perspiring; tongue somewhat coated, but moist; thirst considerable; bowels not open.—Aperient medicines continued.

In the evening I again saw her, and examining the arm, found some inflammation had taken place in the course of the vein. On tracing the vein accurately with my fingers, I found the tenderness increase as I approached the axillary vein, where it empties itself. Pulse 100, strong, and jerking; tongue dry, and rather brown in the centre, but white and moist towards the edges; skin perspiring. Secretion of milk so copious as to produce considerable pain in the breasts. I am disposed to think that the state of the mammæ was the principal cause of these symptoms, as the febrile action was not more than what is frequently observed when the breasts are full and painful; but how far they may have been increased in this instance by the inflammatory action of the vein, is uncertain. Twelve leeches were immediately ordered to the arm in the line of the vein, with directions to apply fomentations to the limb as soon as they dropped off. The lint was taken from the wound, and a poultice applied. These measures, assisted by purgatives, nearly subdued the inflammation and slight tumefaction which had taken place.

Friday.—There was left some tenderness at the upper end of the vein, which had not apparently diminished since my last visit, but which was removed by the application of six more leeches. The secretion of milk was copious, and rather troublesome, as it gave her pain, and required the breasts to be drawn frequently.

It would be useless to detail minutely the subsequent treatment in this case. Suffice it to say, that it consisted of saline aperients, as occasion required, and such other medicines as are usually given in common cases.

October 4th.—The seventh day after the operation she felt quite well, but the wound in the arm was not cicatrised. The medicines were discontinued.

I have to add, that, in consequence of over-exertion in the management of her household affairs, slight inflammation was reproduced in the vein on the 5th October, which is not yet entirely subdued.* This has not excited the system at any time, so as to influence the pulse in any considerable degree. It has not been, at any instance since the inflammation recommenced in the vein, above eighty in the minute.

From this case every thinking mind will, of course, deduce its own inferences.

It might be presumed that, in consequence of the resistance made to the exposure of the vein, and the general restlessness of the patient, she could not have been so much exhausted as I have above stated. These symptoms, however, are common, and are not to be regarded as indicative of strength in such cases, but of some peculiar disturbance of the nervous system, which I am not prepared to explain; and I am borne out in this opinion by their general termination, and by the subsequent assertions of the patient, who said that she had no recollection of having made resistance. This violence appears to me to be allied to that jactitation which sometimes immediately precedes death from flooding, and which I once had an opportunity of witnessing in a woman who died in my presence three hours after the delivery, notwithstanding the most vigorous means were made use of for her preservation.

From the facts above related, it seems to me we learn—

First, That fourteen ounces of blood from the veins of a man may be received in a tumbler, and transferred by means of a syringe, with the greatest facility, to the veins of a woman nearly exhausted by bleeding, without necessarily destroying life, or even, of necessity, producing any dangerous symptoms.

Secondly, From this case, viewed in connexion with that of the woman in Grub-street, operated upon by Dr. Blundell and Mr. Waller;† we might infer that, although symptoms of slight syncope may occur under transfusion, they are not necessarily inseparable from the operation, but accidental; as in this woman,

* Perhaps, where there is choice, it would be better to inject blood in the left arm, instead of the right, which was resorted to in this instance, as the left is less likely to be injudiciously used during convalescence.

† See the last Number of this Journal.

during the operation, effects the very reverse were observed: instead of any appearance of syncope, sudden, and even surprising, invigoration of the system were produced.

Thirdly, As this woman, after the injection of six ounces of blood, felt an increase of strength astonishing even to herself, and as, after the operation, there was a great deal of mammary action, approaching to inflammation, as well as other proofs of a thorough invigoration of the system, it may, I think, be fairly inferred that the injection of fourteen ounces of blood was more than sufficient to supply her wants. It is not unlikely that eight ounces would, under her circumstances, have been enough; and this case, therefore, gives an additional probability to the opinion that the four ounces transfused in the Grub-street case really preserved the life of the patient, as this woman was rather under the middle size.

Fourthly, Two successive cases of transfusion, unattended with dangerous consequences, do not, of course, demonstrate that the operation is wholly secure from danger; but they may raise in us a reasonable hope that such may hereafter be found to be the case. The tenderness and swelling in the line of the cephalic vein, however, though at no period alarming in this woman, must not be lost sight of: but how far it may be accidental, time must decide.

Fifthly, Should the operation hereafter not only prove practicable and easy, but very safe, there are various other cases, besides those of desperate inanition, in which it might be employed with probable advantage. To enumerate these, however, I am not at present prepared; but Dr. B. has suggested to me, in conversation, that perhaps the operation may be found serviceable in cases where too much blood has been taken from the arm, and in those cases where, in consequence of floodings during delivery, various morbid symptoms may be expected, although they be not dangerous to life. Great care, however, must be taken not to abuse the operation, by a too-frequent and unnecessary resort to it.

Sixthly, As this woman survived, it cannot be incontrovertibly asserted that, if the operation had not been performed, she must certainly have sunk. In all these cases there will be room for difference of opinion; but that women do die from uterine bleedings, half an hour, an hour, nay sometimes two, three, or more hours after the hemorrhage has been arrested, is well known: and, I presume, the most sceptical will not deny that fourteen ounces of healthy blood would be sufficient to turn the scale in their favour.

In my humble opinion, it is much to be regretted that this operation has been so long neglected: the deaths of several women from hemorrhage have recently come to my knowledge; and it is probable that hundreds of persons have died since it was first promulgated, whose lives, I think it is not too much to say, might have been preserved by a timely resort to it.

In conclusion, I may say that I feel satisfied it will be found an operation simple and easy; requiring only common care, common caution, and common ability, to perform it with success, as far as the operation is concerned, in all such cases as that which I have above related.

82, *Great Surrey-street, Blackfriars-road;*
*Oct.* 10*th*, 1825.

---

